# Notes on X-Light

**Published:** 1905-02

**Authors:** 


### Bibliography.

Notes on X-Light. By William Rollins. Boston, Mass., MCMIV.

The readers of the International Dental Journal have from time to time been made familiar with "Notes" by William Rollins. All of these have been of an eminently practical character, and especially valuable for their originality. All may not be aware that Dr. Rollins has, aside from his professional labors, a wide reputation as a worker in electrical problems, and of recent years he has been more directly connected with the study of the X-light.

In a former number of this journal the interesting and valuable work of Williams on "The Röntgen Rays in Medicine and Surgery" was reviewed, and much of the apparatus used by that author was from the inventive mind and manipulative mechanical skill of Dr. Rollins.

The author has evidently no love for wordy dissertations. His notes are generally very brief, but in the condensation nothing of importance is omitted.

This large book of four hundred pages and one hundred and fifty-two pages of plates is introduced by the shortest preface coming within the observation of the reviewer. It covers just six lines, and may be copied entire:

"In these notes are recorded some impressions derived from experiments made after the day's work, as a recreation, yet with the hope of learning to design and construct apparatus for my friend, Dr. F. H. Williams, who has done most to show the importance of X-light in medical diagnosis."

The notes are throughout very technical in character, and yet from first to last are of vital interest to the student in X-light problems. The number of these, while comparatively limited, at present are constantly increasing and more and more capable of appreciating an original work of this character.

It seems impossible for those who read the pages of this book not to feel a degree of admiration for the self-sacrificing devotion that has led the author to devote his hours of recreation to experimenting and devising apparatus to render the X-light more efficient and to reduce its manifest dangers. The average individual would recoil from this continuous labor supplementing the exhaustive professional work of the day.

The Notes are not strictly subject matter for review. They have had their origin in the laboratory, and are the product of a mind intensely active and devoted to the subject, hence there is no room for criticism, while there is much for approval from those who are prepared to follow Dr. Rollins and make use of his apparatus to assist them in their work.

These Notes have been originally published in the INTERNATIONAL DENTAL JOURNAL, *Electrical Review, American X-Ray Journal, American Journal of Science,* and *Boston Medical and Surgical Journal.*

Those who have confined their reading upon this subject to its application in dental lesions may have but a limited appreciation of the extended use to which the X-light has been applied. This will be best illustrated by one quotation as typical of this taken from one of the Notes, "On seeing and hearing your heart beat."

"By placing a mirror in proper relation to a luminescent screen, a man can see his own heart beat. By combining the mirror with the instrument (Plate 19), he can both see and hear the heart. A nervous patient whose organs are sound, is sometimes much comforted by being shown through his own eyes that all is well."

The following interesting facts connected with " Vacuum Tube Burns" are worthy of note in this connection. " The first report of burning from a vacuum tube was probably by Hawkes, in the *Electrical Review* for August 12, 1896. Tesla, in the same journal for December 2, 1896, said they were not due to X-rays, but to ozone, and possibly to nitrous acid.

" In the experiments four strong guinea-pigs were used. Two were exposed to X-light under the conditions mentioned in the Notes of February 14 and 28 and March 28, 1901, for protecting them from the effects of ultra-violet light, electric induction, and convection. The others were subjected to the same treatment and handling, except that no X-light was allowed to shine upon them. . . . When a pig was being exposed, his nearest side was fourteen centimetres from the radiant area of the target. It will be observed before the X-light could shine on a pig it passed through two thicknesses of aluminum, the outer one connected with the earth by a metal wire. It should also be remembered Tesla and others have stated a single thickness of aluminum was a protection against ' X-ray burns.' These experiments showed burns could be produced and animals killed by X-light after it had passed through two aluminum screens."

The author follows these with elaborate tables showing the effect on the pigs each day.

In a note on " The Effect of X-Light on the Crystalline Lens," the author states that " No X-light should strike a patient except the smallest beam that will cover the area to be examined, treated, or photographed. . . . An experimenter who works much with X-light should use a non-radiable face-mask, the eye-holes of which are glazed with thick plates of heavy lead glass."

It is impossible to quote extensively from this unique volume, but the temptation is to give the short, crisp, and valuable ideas a wide publicity. Those interested in the subject will find the study of these Notes not only instructive, but interesting, and another evidence, if any be needed, of the wonderful progress made in the development of the X-light since Röntgen startled the world by the announcement of his discovery.

Not the least interesting part of the volume will be found at its close, covering one hundred and fifty-two pages of illustrations of appliances for the use of the X-light. They very perfectly explain the text and the use to which they are to be applied.

The University Press of Cambridge, Mass., is responsible for the printer's part of the work, and this has been very perfectly met. It is artistic throughout in type, press-work, and paper.

